# The hunt for elusive cancer stem cells

**DOI:** 10.18632/oncotarget.17443

**Published:** 2017-04-26

**Authors:** Salvatore Condello, Nkechiyere G. Nwani, Daniela Matei

**Affiliations:** Department of Obstetrics and Gynecology, Northwestern University Feinberg School of Medicine, Chicago, IL, USA

**Keywords:** cancer stem cells, tissue transglutaminase, ALDH, glioblastoma, chemo-resistance

The efficacy of cancer therapeutics remains limited because tumors comprise heterogeneous cell populations, with distinct phenotypic and functional properties. Among those, cancer stem cells (CSCs) represent a small cell subpopulation with self-renewal, differentiation and tumor initiation capacity and have been linked to treatment failure. Unlike differentiated cells, CSCs evade the cytotoxic effects of chemotherapy and radiation contributing to the development of recurrent tumors, which are more aggressive and unresponsive to treatment. The underlying biochemical events that allow this cell population to persist are not fully understood. In this manuscript, Sullivan et al. describe the functions of tissue transglutaminase (TG2) as a survival factor and potential target in mesenchymal glioma stem cells [1].

TG2 belongs to a family of structurally and functionally related proteins that catalyze calcium-dependent transamidation between glutamine residues of a donor protein and primary amino groups of another acceptor protein or polyamine. In addition to catalyzing epsilon-(gamma-glutamyl)lysine isopeptide bonds, TG2 also binds and hydrolyzes guanosine 5′-triphosphate (GTP), acting as GTP-ase linking cell surface receptors to intracellular effectors. Ca2+ and GTP levels finely regulate the two functions of the protein; the crosslinking activity being allosterically activated by high Ca2+ concentrations, and inhibited by high intra-cellular GTP levels [2]. Aberrant TG2 expression was correlated with several pathological conditions, including cancer, and new evidence links TG2 to the CSC phenotype. Cao et al. demonstrated that the transforming growth factor (TGF)-β, a cytokine abundantly secreted in the ovarian cancer microenvironment, activates NF-κB, upregulating TG2 and promoting the formation of cancer cell spheroids [3]. TG2 expression was increased in ovarian CSCs derived from human tumors and defined by co-expression of CD44^+^ and CD117^+^ [3]. TG2 upregulation was also recorded in the highly tumorigenic subpopulation of CD44^+^/CD24^−^ breast CSCs [4], contributing to self-renewal, chemo-resistance and mammosphere-forming capacity and in epidermal CSCs (ECSC) where it promoted sphere formation and tumorigenicity [5].

Here, Sullivan and colleagues describe a new connection between the CSC-marker ALDH and TG2 expression in glioma stem cells (GSCs) [1]. TG2 was found to be robustly expressed in mesenchymal compared to proneural GSCs. Two potent TG2 inhibitors monodansylcadaverine (MDC), a TG2 substrate and competitive inhibitor, and Z-Don, which irreversibly binds to the catalytic site, were used to study the effects of TG2 in GSCs. Both MDC and Z-Don, prevented neurosphere formation and suppressed self-renewal and proliferation of mesenchymal GSCs. TG2 proved to be essential in maintaining the survival of GSCs by preventing apoptosis, particularly under nutrient deprivation. To selectively target the drug-resistant and tumor initiating GSCs, Sullivan and colleagues combined TG2 inhibitors (MDC and Z-Don) with temozolomide and radiation. Combination therapy induced GSCs death with subsequent significant decrease in neurosphere proliferation compared to either radiation or temozolomide treatment alone. Recent data in a different cancer model showed that the GTP-binding activity, associated with a closed conformation of TG2, but not the transamidase activity was required for epidermal CSC (ECSC) survival and spheroid formation [5]. The binding of irreversible inhibitors (NC9, VA4, and VA5) to the catalytic site of TG2 promoted a shift from its closed to an open conformation and led to a reduction in the survival and proliferation of ECSCs [5]. The complex between TG2, fibronectin and integrins has also been proposed as a functional cancer target and inhibitors for this protein-protein interaction are being developed [6]. Altogether these data provide a strong rationale for the potential use of TG2 inhibitors to eliminate CSCs.

**Figure 1:**
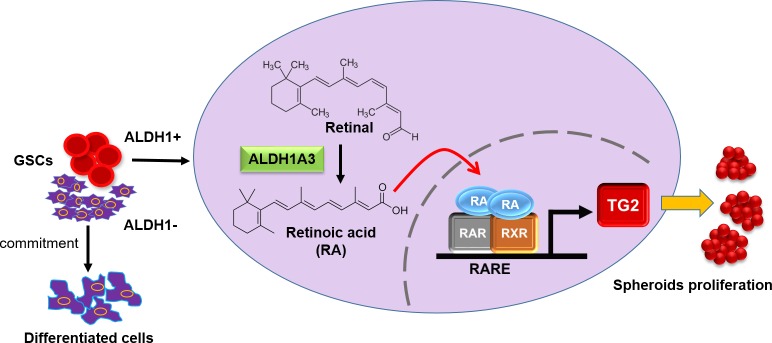
Interaction between ALDH1A3 and TG2 in glioma stem cells

To elucidate the mechanism driving TG2 upregulation in the highly aggressive mesenchymal GSCs, the authors explored the relationship between the enzyme and the stem cell marker ALDH1A3. Aldehyde dehydrogenases (ALDHs) comprises a family 19 evolutionary conserved NADP(^+^)-dependent enzymes that regulate cellular detoxification and tissue homeostasis by catalyzing the oxidation of endogenous and exogenous aldehydes into corresponding acids [5]. High ALDH activity and expression has been associated with increased cancer cell aggressiveness, metastatic potential, pluripotency and chemo-resistance in several cancer types. [7] Recent studies implicate ALDH-mediated regulation of retinoic acid (RA), reactive oxygen species (ROS) and aldehydes in CSCs maintenance. Among the isoenzymes, ALDH1 plays the most prominent role in CSCs, being deregulated in both hematologic and solid tumors [8]. RA is a ligand for nuclear RA (RARs) and retinoid X receptors (RXRs) and RA responsive genes, are involved in critical biological processes such as embryonic development, morphogenesis, growth, and differentiation. *TGM2* is a known RA target and the authors show here a robust correlation between ALDH1A3 and TG2 expression in patient-derived GSC, glioblastoma, and astrocyte cell lines [1]. TG2 expression inhibited by ALDH1A3 gene knockdown or chemical inhibition using DEAB in mesenchymal GSCs was rescued by addition of RA. Furthermore, overexpression of ALDH1A3 induced TG2, whereas a catalytically inactive form of ALDH1A3 (C314A) was ineffective, supporting a link between retinoid signaling facilitated by ALDHs with TG2 induction.

These data and other emerging reports support that the multi-functional TG2 is enriched in CSCs and represents a robust therapeutic target. Whether enzymatic, GTPase, or protein-protein inhibitors affecting TG2's interactions with partner proteins should be used, depends on cell type context and further in depth analysis of the specific functions of TG2 in cancer stem cells. The ultimate goal remains to eliminate recalcitrant cancer cells resistant to standard treatment.
